# Editorial: Acute-on-chronic liver failure: systemic inflammation and immunosuppression

**DOI:** 10.3389/fimmu.2023.1260749

**Published:** 2023-08-15

**Authors:** Olaf Tyc, Yu Shi, Yu-Chen Fan, Jonel Trebicka, Xiaogang Xiang

**Affiliations:** ^1^ Medical Clinic 1, University Hospital, Goethe University Frankfurt, Frankfurt, Germany; ^2^ State Key Laboratory for Diagnosis and Treatment of Infectious Diseases, Collaborative Innovation Center for Diagnosis and Treatment of Infectious Disease, National Clinical Research Center for Infectious Diseases, The First Affiliated Hospital, Zhejiang University School of Medicine, Hangzhou, China; ^3^ Department of Hepatology, Qilu Hospital, Shandong University, Jinan, China; ^4^ Department of Internal Medicine B, University Hospital of Münster, Münster, Germany; ^5^ European Foundation for the Study of Chronic Liver Failure (EF-CLIF), Barcelona, Spain; ^6^ Department of Infectious Diseases, Ruijin Hospital, School of Medicine, Shanghai Jiao Tong University, Shanghai, China

**Keywords:** acute-on-chronic liver failure, systemic inflammation, immunosuppression, pathophysiological mechanisms, chronic liver disease

## Introduction

ACLF is a severe and often fatal condition that occurs in individuals with underlying chronic liver disease when acute events precipitate its development (1). Due to the lack of therapies for ACLF, except for liver transplantation, there is a critical need to investigate its pathophysiological mechanisms (2). It is well-established that systemic inflammation plays a significant role in driving ACLF (3). However, there is limited understanding of how systemic inflammation develops and how it affects organ functions. Additionally, immunosuppression, another immune dysfunction observed in ACLF, contributes to bacterial infections and worsens the overall condition (4). Unfortunately, little is known about the development of immunosuppression during ACLF and its underlying molecular mechanisms. Consequently, ACLF remains a major challenge for clinicians and researchers. The following articles present interesting findings in the field of Acute-on-chronic Liver Failure (ACLF).

## Editorial on the Research Topic acute-on-chronic liver failure: systemic inflammation and immunosuppression

The first part of this Research Topic consists of investigations using biosamples from patients, which provide biomarkers and insights in potential immunological effects of specific drugs. The study of Sánchez-Rodríguez et al. investigates the role of CD5L (a macrophage anti-inflammatory protein) and the specialized pro-resolving lipid mediators (SPMs) in the pathogenesis of acute-on-chronic liver failure (ACLF). The study revealed a progressive loss of circulating EVs as the disease progressed. Moreover, the content of CD5L in EVs exhibited differential changes during disease progression, with the highest levels observed in AD and a subsequent decrease in ACLF. This observation indicates a potential role of CD5L in regulating immune cell activation and the systemic inflammatory response. Hereby, monocytes are key cell type. Tong et al. reported about the effect of granulocyte colony stimulating factor (G-CSF) on switching the M1 pro-inflammatory state to M2 anti-inflammatory state of monocytes in ACLF patients from a randomized controlled trial. G-CSF therapy not only induces M1/M2 phenotype of monocyte but also attenuates pro-inflammatory cytokine secretion, but do not influence phagocytosis or oxidative burse capacity in patients with HBV-ACLF, which may lead to resolution of inflammation and ACLF recovery in selected patients. Maheshwari et al. analysed defects in phagocytic and oxidative burst capacity in ACLF monocytes in an animal model of ACLF. MSC therapy may correct the energy supply and eventually ameliorate hepatic injury and promote liver regeneration. Moreover, drug-induced liver injury (DILI) may also precipitate ACLF in few cases, which is shown in the prospective cohort study of Wang, M.-G. et al. The authors investigated the mechanisms underlying DILI due to anti- tuberculosis drugs (ATB) on diagnosed tuberculosis (TB) patients (Wang, M.-G. et al.). The authors analyzed urinary metabolic and microbial samples. The resulting data were submitted to machine learning techniques, which allowed the development of a prediction model for ATB-DILI based on the obtained metabolomics, microbiome, and clinical data.

The second topic was about to perform manipulations on disease model animals to provide insights into pathophysiological mechanisms of ACLF pathogenesis. MicroRNA (miRNA) is known to bind to specific sequences in target mRNAs and thereby silence gene translation. Tao et al. tested the therapeutic efficacy of miR-125b-5p supplement, which was depleted in the liver of HBV-ACLF patients in a model of liver failure induced by lipopolysaccharide (LPS) and D-galactosamine (D-GalN). The results showed that miR-125b-5p alleviated mouse ALF, most probably *via* Kelch-like ECH-associated protein 1 (Keap1) repression and up-regulation of the expression of nuclear factor (erythroid-derived 2)-like 2 (Nrf2) and Heme oxygenase-1 (HO-1).

By contrast, Qu et al. focused on the role of PEBP4 pathway, and using PDTC and TAK-242, that selectively inhibits the activity of NF-κB and TLR4, which can partially reverse the detrimental effect of PEBP4 depletion. The work of Qu et al. provides another candidate target for drugs that are partly in clinical testing as ACLF therapy. This part of the Research Topic is rounded-up by the work of Huang et al. investigating the role of mitophagy, in the pathogenesis of acute liver injury (ALI) caused by heat stroke (HS) which is a fatal form of heat injury. This study revealed a cytosolic p53-mediated impaired mitophagy in the pathogenesis of HS-ALI and highlights pharmacologic induction of mitophagy by inhibiting cytosolic p53 as a promising therapeutic approach for HS-ALI treatment. Also the work of Wang, J. et al. tested the therapeutic efficacy of phenethyl isothiocyanate (PEITC), a natural compound extracted from cruciferous vegetables, in an concanavalin A (ConA)-induced acute liver injury model and carbon tetrachloride (CCl4)-induced chronic liver injury model. Mechanically, PEITC inhibited hepatocyte pyroptosis by interacting with cysteine 191 of GSDMD with its specific structure –N=C=S, which may be a candidate drug for treating ALI.

The Research Topic also collected three up-to-dated literature reviews. The review of Qiang et al. incorporates the current research status related to ACLF and sheds light on the immune mechanisms underlying the development and progression of acute-on-chronic liver failure (ACLF). The authors discuss that the understanding of ACLF and its definition has been a subject of controversy discussion and definition differences among the liver-related research communities worldwide. In their review, the authors highlight the immune pathogenesis of ACLF and the importance of pathogen-associated molecular patterns (PAMPs) and damage-associated molecular patterns (DAMPs) in ACLF. Furthermore, the review of Hazrati et al. summarizes the mechanisms underlying the protective and therapeutic effects of MSCs and MCSs- derived extracellular vesicles (EVs) in liver diseases, involving differentiation into hepatocyte-like cells, inhibition of apoptosis and inflammation, promotion of growth factors production and hepatocyte proliferation, suppression of hepatic stellate cells (HSCs) and tissue-damaging immune cells.

Finally, the review of Ye et al. summarizes the evidences on the use of glucocorticoids in treating ACLF and ALF. The review article emphasizes the etiology of liver failure in evaluating glucocorticoid efficacy. The benefit of glucocorticoid therapy seems to be definite in AIH-induced liver failure, while controversial in HBV or drug-related ACLF or ALF. The authors highlighted the potential impact of dosing and timing of glucocorticoids use on the survival benefit and the lack of specific biomarkers to precisely guide the optimized use of glucocorticoids in these patients.

## Author contributions

OT: Conceptualization, Writing – original draft. YS: Writing – original draft, Writing – review & editing. Y-CF: Investigation, Writing – original draft. JT: Data curation, Writing – original draft. XX: Formal Analysis, Writing – original draft.
